# Fertility-sparing strategy in a rare case of uterine adenosarcoma and consecutive spontaneous pregnancy and livebirth

**DOI:** 10.1007/s00404-026-08443-x

**Published:** 2026-06-23

**Authors:** E. Ziegler, J. Altmann, W. D. Schmitt, S. Mechnser, J. Sehouli, E. Roser

**Affiliations:** 1 Department of Gynecology With Center of Oncological Surgery and Endometriosis Center, Charité-University Hospital, Charitéplatz 1, 10117 Berlin, Germany; 2 Department of Pathology, Charité-University Hospital Berlin, Charitéplatz 1, Berlin, Germany

**Keywords:** Uterine adenosarcoma, Fertility-sparing, Fertility preservation, Spontaneous pregnancy

## Abstract

**Introduction:**

We present the rare case of a 29-year-old patient with uterine adenosarcoma who received fertility-sparing treatment, subsequently conceived spontaneously, and gave birth to a healthy infant.

**Case:**

In August 2022, the nulliparous patient presented with acyclic uterine bleeding. Diagnostic hysteroscopy and targeted resection of a polyp located at the cervicouterine junction revealed uterine adenosarcoma without sarcomatous overgrowth (FIGO stage T1a). Imaging confirmed no residual tumor or metastasis. A fertility-sparing management strategy was chosen, avoiding hysterectomy but involving close oncological surveillance with quarterly MRI scans. During follow-up, a concurrent diagnosis of symptomatic endometriosis introduced therapeutic challenges. The patient spontaneously conceived in 2023 and delivered a healthy infant by cesarean section at term in 2024. Subsequent hysteroscopy and imaging in 2025 showed no evidence of recurrence, therefore fertility-preserving management was continued.

**Patient Perspective:**

A short narrative of the patient’s perspective on her initial diagnosis, possible fertility loss, and the emotional turmoil of pregnancy after uterine adenosarcoma is presented.

**Discussion:**

Treating young patients diagnosed with uterine adenosarcoma poses a challenge to the treating physician due to the lack of guidelines and evidence regarding fertility-preserving treatment of this rare tumor. This report adds to the limited literature on successful pregnancy after uterine adenosarcoma and discusses indications and limitations of fertility-sparing treatment.

**Conclusion:**

Fertility preservation may be possible in selected early-stage uterine adenosarcoma cases without high-risk features such as sarcomatous overgrowth or myometrial invasion, with thorough counseling and strict follow-up. Further research is needed to develop standardized protocols and improve management in this context.

## Take-home message

**Table:** 

What does this study add to the clinical work? The management of rare gynecological malignancies presents a significant challenge for clinicians and becomes even more complex when fertility preservation is desired. Treatment planning should ensure oncological safety while exploring fertility-sparing approaches.	

## Introduction

Uterine adenosarcoma (UAS) is a rare gynecological malignancy [1] composed of biphasic tumor proliferation with a malignant mesenchymal component showing a phyllodes-like architecture and a benign epithelial component. In general, UAS occurs as an exophytic and polypoid mass, filling the uterine cavity and sometimes protruding through the cervical os [2].

It most commonly occurs in postmenopausal women, with peak incidence in the sixth and seventh decades of life, but it has been reported across a wide age range [3]. The treatment of choice is total hysterectomy and possibly—although evidence regarding benefit is not sufficient—bilateral salpingo-oophorectomy [4, 5]. European Guidelines suggest pursuing bilateral salpingo-oophorectomy in postmenopausal women [6], whereas for premenopausal women the potential risk of increased recurrence [7] has to be weighed thoroughly against the possibility of fertility preservation. When considering fertility-sparing treatment strategies for younger patients diagnosed with UAS, the histologic factors associated with poor prognosis are key indicators for patient outcome [8, 9]. These include sarcomatous overgrowth, defined as a high-grade sarcomatous component comprising at least 25% of the tumor, along with deep myometrial and lymphovascular invasion [10]. Recurrence occurs in 15–25% of adenosarcomas without sarcomatous overgrowth but rises sharply to 45–70% when overgrowth is present. The mortality rate for typical adenosarcoma ranges from 10 to 25% but increases significantly—up to 75%—in cases with sarcomatous overgrowth [11]. In general, fertility-preserving strategies usually refer to well-circumscribed polypoid lesions that can be completely resected by hysteroscopy. German and European guidelines recommend at least 6 months of treatment with medroxyprogesterone acetate (MPA) 160 mg/d following R0 resection achieved via hysteroscopy.

This case report presents a 29-year-old woman with a desire to preserve fertility who was diagnosed with UAS. Initially advised to undergo total hysterectomy, she sought a second opinion and subsequently received fertility-sparing treatment. She later conceived spontaneously and delivered a healthy infant. The patient’s perspective on the care received is summarized in a short narrative.

A review of the literature concerning fertility-sparing management of UAS is presented (see Table 1), with a focus on reported cases of successful pregnancy following conservative treatment of UAS at stage 1A.

**Table 1:** Cases of pregnancy and livebirth after fertility-preserving treatment in patients with uterine adenosarcoma stage 1 A (based upon table presented by Zizolfi et al. [12])

Authors	Age at diagnosis	Clinical presentation	Treatment	Modality of follow-up	Age at conception	Type of conception	Pregnancy outcome	Oncological outcome at the publication
Lee et al., 2017	33	NS	HSC-mass excision + MPA for 3 months	NS	34	Spontaneous	Full-term vaginal delivery	NED after 77 months
Goh et al., 2018	21	Heavy vaginal bleeding, endometrial polyp	HSC-Polypectomy	Interval ultrasound	24	Spontaneous	Full-term vaginal delivery	Recurrence after 8 years, therefore TLH + BSO + bilateral PLD; post-TLH NED after 43 months
L'Heveder et al., 2019	18	Heavy vaginal bleeding, endometrial polyp	HSC-Polypectomy	Biannual pelvic ultrasound, HSC + endometrial biopsy; annual pelvic MRI after pregnancy, annual ultrasound, HSC + endometrial biopsy	30	IVF	Preterm vaginal delivery	NED for 20 years, then TLH at patient's request
Zizolfi et al., 2021	23	NS	HSC-mass excision + MPA for approx. 24 months, discontinued due to desire for conception	Biannual HSC + endometrial biopsy; annual pelvic MRI	27	Spontaneous, after one failed IVF cycle	Full-term vaginal delivery	NS

*BSO* bilateral salpingo-oophorectomy, *HSC* hysteroscopy, *IVF* in vitro fertilization, *MPA* medroxyprogesterone acetate, *MRI* magnetic resonance imaging, *NED* no evidence of disease, *NS* not specified, *PLD* pelvic lymph node dissection, *TLH* total laparoscopic hysterectomy

## Methods

This case report was written using the patient’s data extracted from the SAP patient documentation software, including medical reports, histopathological and immunohistochemical analysis reports and surgery documentation. A retrospective analysis of the existing documentation was conducted, using in-house documentation as well as documents provided by the outpatient gynecologist and the initially treating hospital.

To incorporate the patient’s perspective, a comprehensive semi-structured interview was conducted in June 2025. The narrative was transcribed and returned to the patient for validation to ensure accuracy and authenticity. The patient has provided informed consent for the publication of this case report, including the narrative presented, confirming that it accurately displays her experience.

The literature review included in the discussion section was conducted using the PubMed database, employing search terms, such as “uterine adenosarcoma” and “fertility preservation”. Given the rarity of uterine adenosarcoma and the scarcity of robust clinical studies, the review focused on published case reports documenting live births following fertility-preserving treatment in patients with UAS. Relevant cases were identified and systematically analyzed. The findings are presented in Table 1, which was adapted and expanded based on the structure of a previously published summary table by Zizolfi et al. in a case report from 2021[12].

## Case report

In January 2022, the 29-year-old patient presented at her gynecologist with complaints of acyclic uterine bleeding despite neither prior nor ongoing hormonal treatment, along with an irregular menstrual cycle. The patient was nulliparous and had no significant medical history.

**Fig. 1:**
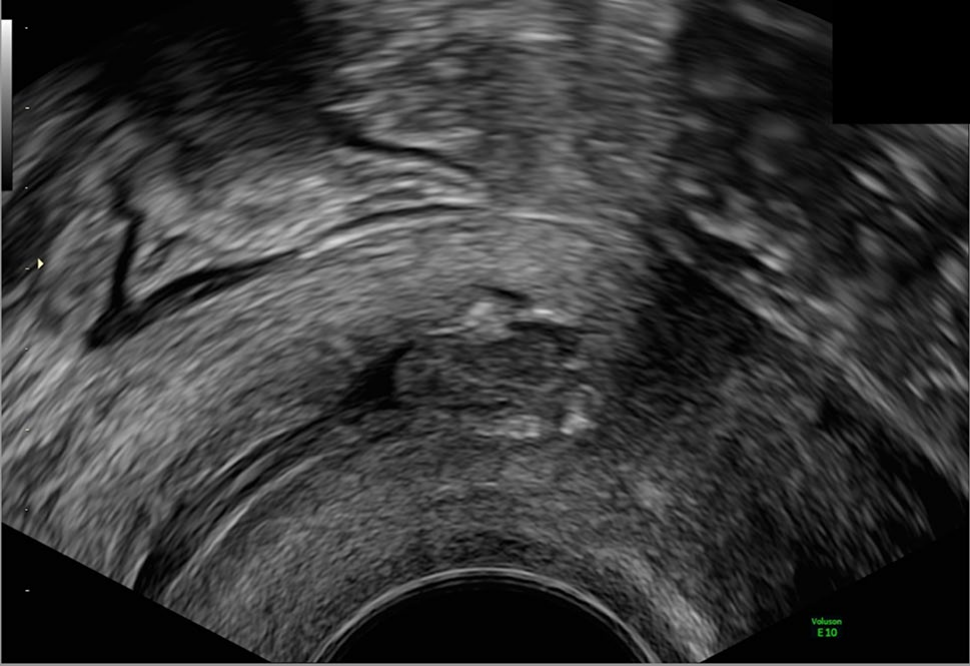
HYPERLINK "sps:id::fig1||locator::gr1||MediaObject::0" :Transvaginal sonogram shows a polyp located at the cervicouterine junction

**Fig. 2:**
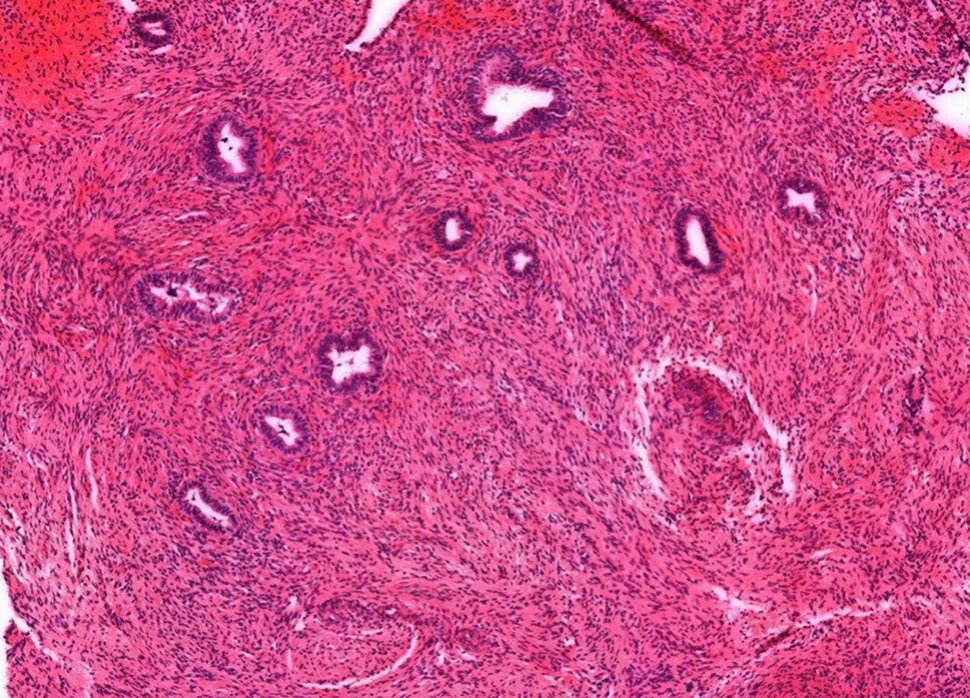
Histopathological image showing malignant stromal tissue alongside benign epithelial glands

Clinical examination revealed a polyp located at the cervicouterine junction, measuring 16 × 14 × 9 mm in sonography (see Fig. 1). A diagnostic hysteroscopy with targeted polyp resection was performed. Histopathological examination revealed a UAS without evidence of sarcomatous overgrowth (see Fig. 2).

Microscopic evaluation of the resected material showed fragments of endometrium from the uterine corpus with mildly wavy glands and occasional subnuclear vacuolization. Multiple areas exhibited a lesion with phyllodes-type architecture and a polypoid configuration. Stromal condensation was observed in small foci beneath the surface epithelium and around glands embedded within the stroma. Within these condensed stromal areas, up to three mitotic figures per ten high-power fields were identified. Immunohistochemical analysis revealed that the atypical glandular complexes demonstrated a slightly increased reactivity to CD10 within the peri-glandular stromal cuff. The lesion exhibited mildly elevated proliferative activity, with a Ki-67 index of approximately 4%. Stromal cells showed uniform expression of estrogen receptors (100%, moderate intensity) and progesterone receptors (100%, moderate to strong intensity), along with positive staining for WT1.

Due to the patient’s young age, a secondary consultative pathological examination was performed, yielding the same results. Therefore, the diagnosis of adenosarcoma of the uterus without sarcomatous overgrowth (FIGO T1a) was confirmed.

The patient was advised to undergo a total hysterectomy as soon as possible. Due to her desire to preserve fertility, the patient sought a second opinion at our clinic regarding fertility-sparing treatment options.

Tumor staging with thoracic and abdominal CT and abdominal–pelvic MRI showed no evidence of metastases or residual tumor. Another hysteroscopy was performed, and endometrial biopsies of the isthmo-cervical junction and the corpus uteri were taken. No remaining tissue of the previously known adenosarcoma was detected.

Given the early clinical stage of the adenosarcoma, the absence of sarcomatous overgrowth, and the patient’s wish to preserve fertility, a conservative treatment approach was recommended. This strategy involved deferring total hysterectomy in favor of close oncological surveillance, including abdominal and pelvic MRI every three months. During this period of close surveillance, a diagnosis of endometriosis with a left ovarian endometrioma, causing severe dysmenorrhea, was confirmed. This introduced a complex clinical scenario: managing oncological follow-up post-uterine adenosarcoma while addressing symptomatic endometriosis—a combination for which scientific data and clinical guidelines remain limited.

**Fig. 3:**
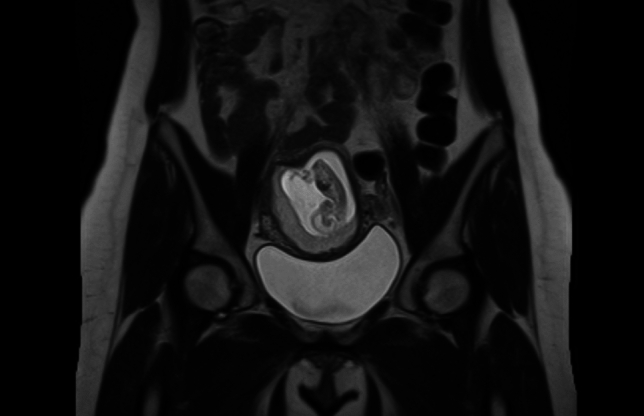
Follow-up MRI image taken while the patient was pregnant

In June 2023, the patient conceived spontaneously (see Fig. 3). Throughout the pregnancy, regular sonographic examinations and a pelvic MRI revealed no signs of recurrence or metastases. In March 2024, due to marginal placental insertion and umbilical cord entanglement, a primary cesarean section was performed at 39 weeks’ gestation. Intraoperative evaluation of the uterus during the cesarean section did not reveal any abnormalities. The patient delivered a healthy infant.

The interdisciplinary tumor board discussed the option of a secondary hysterectomy following the patient’s recent childbirth. However, the patient expressed a strong desire to preserve fertility with the hope of conceiving again. In 2025, a third hysteroscopy with fractional curettage was performed, revealing endocervical and ectocervical mucosa free of dysplasia. Pelvic MRI for oncological staging was unremarkable, showing no signs of malignancy or significant pathology. A definitive oncological recommendation for hysterectomy was issued by the treating physicians; however, after shared decision-making with the patient, fertility-preserving management was continued considering her sustained remission for over two years and the assessed low risk of tumor recurrence. Due to her concurrent diagnosis of endometriosis, the recommended treatment option for the patient is a course of Linzagolix, beginning with a higher dose followed by a dose reduction after three months of therapy. Additionally, the option of fertility preservation through oocyte cryopreservation is being considered.

The patient is currently under intensive oncological monitoring, with pelvic and abdominal MRI and sonography performed alternately at three-month intervals through December 2025, alongside regular gynecological examinations. Should remission be maintained for three years post-diagnosis, follow-up will transition to semiannual gynecological evaluations with transvaginal and abdominal ultrasound.

## Patient perspective

The following narrative summarizes insights shared by the patient during a comprehensive interview, reflecting her experience from initial diagnosis through treatment and post-treatment decision-making. The patient has confirmed that the narrative accurately reflects her experience.

*I first learned of my diagnosis—uterine adenosarcoma—following an initial hysteroscopy, when my gynecologist urgently requested to see me for a consultation. While I suspected that something serious had been found, the confirmation of a rare cancer and the immediate recommendation for hysterectomy were deeply shocking. The idea that I would lose my uterus and therefore the ability to have children had never crossed my mind. My partner and I were devastated. It felt like a double shock: comprehending a cancer diagnosis while at the same time mourning the loss of fertility, of the possibility of having children.*

*My partner and I began researching and ultimately sought a second opinion at a specialized sarcoma center. While my gynecologist supported this decision and facilitated contact with the center, the physicians at the initial hospital were highly dismissive. They discouraged a second opinion, emphasized the urgency of surgery, and showed little empathy when I inquired about the possibility of preserving my uterus, even temporarily. Attempts by the sarcoma center to collaborate with the initial team were met with very limited feedback. I was disappointed by that.*

*In contrast, the sarcoma center offered a more individualized and empathetic approach. It validated my desire to preserve fertility and proposed a second biopsy and a full review by an interdisciplinary tumor board before making definitive decisions. Although this period of uncertainty was emotionally challenging, the decision to seek a second opinion was worth it, because in the end, I was able to keep my uterus. I became pregnant a few months later.*

*The pregnancy was both joyful and anxiety-provoking. I experienced a number of complications, like bleeding, gestational diabetes, and a marginal placental insertion, which necessitated a planned cesarean section. I remained fearful of recurrence throughout, but received attentive, close care that helped me feel supported. All the while, I never really allowed myself to be fully happy about the pregnancy until seeing my healthy son. It was an overwhelming and emotional moment.*

*Currently, my partner and I are weighing the option of a secondary hysterectomy against the option of watchful waiting and possibly another pregnancy. While I am worried about the risk to my health, which is of course further deepened by delaying the hysterectomy, my doctors have reassured me that close surveillance remains a safe approach while we make these considerations. The diagnosis of endometriosis, which came unexpectedly during follow-up, makes this decision even more difficult, as I am told it may also complicate a possible second pregnancy.*

*Looking back, I would advise others in similar situations to try and keep calm when first facing their diagnosis and not to research their disease in online forums, which for me only made my fear worse. Most importantly, I encourage healthcare professionals to respect and incorporate patients’ wishes to preserve fertility into the treatment discussion, communicate with empathy, and collaborate openly with centers of expertise. Recognizing the value of second opinions and placing patient-centered care above institutional interests can make a crucial difference for patients’ health and trust.*

## Discussion

The central dilemma in managing UAS in young patients lies in balancing oncologic safety with the desire to preserve fertility, requiring careful risk assessment and individualized treatment planning. Due to the scarcity of evidence, such rare cases should be managed at specialized treatment centers, where interdisciplinary tumor boards can provide expert guidance.

This case presents a rare clinical challenge. Given the patient’s young age and strong desire to preserve fertility, a conservative treatment approach was considered. As the tumor was staged T1a and confined to the polyp with no evidence of sarcomatous overgrowth, deep myometrial invasion, or lymphovascular involvement, a fertility-sparing strategy was deemed appropriate. The patient received multiple hysteroscopies with targeted polyp resection and follow-up endometrial biopsies. As current evidence does not support a benefit from adjuvant systemic therapy or radiotherapy in UAS [13], these treatments were not administered.

Complicating the clinical picture further, the patient was diagnosed during oncological surveillance with endometriosis, including ovarian endometriomas causing severe dysmenorrhea.

Endometriosis has been increasingly recognized as a potential risk factor for the development of adenosarcoma, particularly in extrauterine cases arising within the abdomen or pelvis. Several reports document extrauterine adenosarcomas originating from sites of histologically confirmed endometriosis, supporting the concept that ectopic endometrial tissue can undergo malignant transformation, although a definitive causal relationship has not been established [14].

This introduces an additional layer of complexity as treatment for endometriosis must now be balanced with oncologic safety and the goal of future pregnancy. Potential treatment options include medroxyprogesterone acetate (MPA) to inhibit endometriosis progression and potentially prevent tumor recurrence [15]; however, it was not administered in this case due to its contraceptive effects and the patient’s desire to conceive. The recommended treatment option for the patient is a course of Linzagolix to treat her endometriosis. However, both aforementioned treatment options are not compatible with attempts to conceive and would necessitate a temporary cessation of medication, possibly leading to a worsening of endometriosis symptoms during the treatment-free interval. While undergoing treatment for endometriosis, close monitoring of the patient’s fertility markers, such as Anti-Müllerian Hormone, is recommended. Given the rarity of this clinical scenario, further research is needed, particularly to explore potential links between gynecologic tumors and endometriosis, and how these conditions may influence each other’s development and management.

In this case, the patient conceived spontaneously just a few months after tumor resection and subsequently delivered a healthy infant approximately one year following the initial diagnosis. To date, to the best of our knowledge, only four other cases have been reported of patients with UAS who underwent fertility-sparing treatment and successfully delivered healthy infants [12, 16–18]. This case report presents key details on diagnosis, treatment, and both oncologic and fertility outcomes, as showcased in Table 1, adapted from Zizolfi et al. [12]. In all four reported cases, initial surgical management involved either hysteroscopic polypectomy or mass excision. Two patients received adjuvant medroxyprogesterone acetate therapy. Three conceived spontaneously, while one achieved pregnancy through IVF. Only one patient experienced recurrence after 8 years of follow-up and was subsequently managed with total laparoscopic hysterectomy, bilateral salpingo-oophorectomy, and pelvic lymph node dissection.

It is important to note that in all presented cases, including this one, there was no evidence of sarcomatous overgrowth, and the tumors were consistently staged as T1a, making them appropriate candidates for a conservative treatment strategy. Since oncologic outcomes are well established only for total hysterectomy [13], the risks of fertility-sparing treatment in UAS must be carefully discussed and evaluated with the patient, with thorough informed consent being essential. A thorough fertility assessment and a discussion of the patient’s realistic chances of conception should be considered. Finally, an interdisciplinary discussion among gynecological oncologists, surgeons, and reproductive medicine specialists should be conducted. To ensure a collaborative and participatory decision-making process, a strong clinician–patient relationship built on mutual trust is essential. The possibility of fertility loss can have a profound psychological impact on young patients, highlighting the need for compassionate counseling, supportive care, and thoughtful consideration of the patient’s wish to preserve fertility—even in the context of a serious diagnosis [19].

Based on the literature, total hysterectomy without morcellation remains the standard treatment for gynecological adenosarcoma [14]; however, in carefully selected cases, fertility-preserving approaches may be feasible without a clearly increased risk of recurrence or significant difference in overall survival, given the current limited evidence [5, 9].

When pursuing fertility-sparing treatment, close oncological monitoring is essential. Given the potential for late recurrences after primary surgery, prolonged surveillance is warranted [20]. Currently, no standardized guidelines exist for optimal follow-up strategies. However, regular hysteroscopies with endometrial biopsies, transvaginal ultrasound, and periodic MRI and, if necessary, CT imaging are commonly utilized in the literature, as was done in the present case.

Following primary fertility-preserving treatment, a secondary hysterectomy may be indicated after completion of family planning [14]. In our case, the patient’s ongoing desire to preserve fertility presents a continued challenge, requiring careful oncologic monitoring while maintaining reproductive potential. In addition, regarding the patient’s first pregnancy and when considering future pregnancies, it is important to account for the potential impact of endometriosis on the course of gestation. Emerging evidence suggests a possible association between endometriosis and placental complications [21, 22].

As illustrated in the patient perspective section included in this case report, the rarity of this clinical situation can lead to disparities in treatment recommendations between hospitals and individual clinicians. To optimize patient outcomes, it is essential that care be provided at a specialized sarcoma center with the necessary expertise and resources to manage such unprecedented and complex cases. Equally important is consistent and collaborative teamwork among treating clinicians, from the initial presentation at an outpatient clinic through to long-term oncological follow-up [23]

## Conclusion

UAS poses a significant clinical challenge due to its rarity and the resulting lack of robust evidence and standardized treatment guidelines. This complexity is further heightened when UAS occurs in a young patient who desires fertility preservation and does not wish to undergo the standard treatment of total hysterectomy.

Fertility preservation may be feasible in select cases of UAS, provided that certain prognostic factors are carefully assessed, and that stringent follow-up can be ensured. The most critical considerations in evaluating eligibility for conservative management include the presence of high-grade sarcomatous overgrowth and myometrial invasion, both of which are associated with poorer outcomes. In light of the rarity of this tumor entity, a secondary pathological assessment is warranted to ensure correct diagnosis and staging. In the absence of these high-risk features, a fertility-sparing approach may be cautiously considered, contingent upon comprehensive patient counseling regarding the associated oncological risks and the need for diligent, long-term surveillance. To ensure close and comprehensive oncological follow-up, the treatment of such rare and complex cases should, if possible, be conducted at specialized sarcoma centers, where informed decision-making and patient-centered care are essential.

To advance care for this rare clinical scenario, further research is essential. Expanding the evidence base and developing standardized protocols for fertility preservation in UAS will be key to supporting safe and personalized treatment planning.

## Data Availability

No datasets were generated or analyzed during the current study.
